# Calpain and PARP Activation during Photoreceptor Cell Death in P23H and S334ter Rhodopsin Mutant Rats

**DOI:** 10.1371/journal.pone.0022181

**Published:** 2011-07-12

**Authors:** Jasvir Kaur, Stine Mencl, Ayse Sahaboglu, Pietro Farinelli, Theo van Veen, Eberhart Zrenner, Per Ekström, François Paquet-Durand, Blanca Arango-Gonzalez

**Affiliations:** 1 Institute for Ophthalmic Research, Centre for Ophthalmology, University of Tübingen, Tübingen, Germany; 2 Department of Ophthalmology, Clinical Sciences Lund, University of Lund, Lund, Sweden; Alcon Research, Ltd., United States of America

## Abstract

Retinitis pigmentosa (RP) is a heterogeneous group of inherited neurodegenerative diseases affecting photoreceptors and causing blindness. Many human cases are caused by mutations in the rhodopsin gene. An important question regarding RP pathology is whether different genetic defects trigger the same or different cell death mechanisms. To answer this question, we analysed photoreceptor degeneration in P23H and S334ter transgenic rats carrying rhodopsin mutations that affect protein folding and sorting respectively. We found strong activation of calpain and poly(ADP-ribose) polymerase (PARP) in both mutants, concomitant with calpastatin down-regulation, increased oxidative DNA damage and accumulation of PAR polymers. These parameters were strictly correlated with the temporal progression of photoreceptor degeneration, mirroring earlier findings in the phosphodiesterase-6 mutant *rd1* mouse, and suggesting execution of non-apoptotic cell death mechanisms. Interestingly, activation of caspases-3 and -9 and cytochrome c leakage—key events in apoptotic cell death—were observed only in the S334ter mutant, which also showed increased expression of PARP-1. The identification of the same metabolic markers triggered by different mutations in two different species suggests the existence of common cell death mechanisms, which is a major consideration for any mutation independent treatment.

## Introduction

Retinitis Pigmentosa (RP) is a genetically and clinically heterogeneous group of neurodegenerative diseases. Symptoms of RP include night blindness, gradual loss of peripheral visual field and eventual loss of central vision, caused by initial death of rods and by secondary degeneration of cones, respectively [1]. The disease prevalence is about 1/4000 [2] with autosomal dominant (ADRP; 30–40%), autosomal recessive (56–60%) or X-linked (5–15%) inheritance patterns [3]. To date, over 45 genes have been identified to be responsible for RP (RetNet; http://sph.uth.tmc.edu/Ret-Net/).

Of particular importance are RP-causing mutations in the rhodopsin molecule. Rhodopsin is unique in the sense that it has distinct regions that are specialized for light capturing, initiation of the phototransduction cascade, rapid deactivation after light absorption, as well as proper folding and sorting within the photoreceptor membranes [4]. More than 100 different mutations have been documented in the rhodopsin gene, accounting for 30–40% of ADRP cases and highly variable disease phenotypes depending on the location of the mutation [5]. The high prevalence and heterogeneity of rhodopsin mutations make it interesting to study the corresponding neurodegenerative mechanisms, not the least for the development of therapies.

The substitution of histidine for proline in the 23^rd^ amino acid (P23H) at the rhodopsin N-terminus has been observed in approximately 12% of ADRP patients [6] and is responsible for the majority of rhodopsin-related RP cases. These patients show a relatively mild clinical progression [7]. On the other hand, C-terminal mutations generally have a more severe clinical phenotype than those in other parts of the molecule [5]; possibly because this domain is important (i) for rhodopsin sorting to rod outer segments and (ii) for rhodopsin phosphorylation and binding of arrestin, two steps essential for deactivation of the protein after light absorption [8]. Hence, a mutation that interferes with both sorting and shutoff of rhodopsin will profoundly change photoreceptor physiology with bearings on cell death [9].

The human mutations are faithfully reproduced in animal models such as the P23H or the S334ter rat. Both models express the respective mutant rhodopsins and, similar to what is seen in human patients, the degeneration in the N-terminal P23H mutant progresses more slowly than in the C-terminal S334ter mutant [10]. In P23H rats rhodopsin appears to be misfolded in the endoplasmic reticulum (ER) [11], which in turn may cause ER stress and unfolded protein response leading to photoreceptor death [12]. In S334ter rats the opsin is truncated at its C-terminus and lacks the last 15 amino acid residues. Similar to the analogous human Q334ter, this RP mutation displays fast degeneration [13]. Furthermore, the rhodopsin transgenic models exhibit protein folding defects and intracellular aggregates, characteristics also present in many neurodegenerative diseases including Alzheimer and Parkinson [14].

Regardless of the primary genetic defect, photoreceptors in RP eventually meet the common fate of cell death, which is likely to involve the action of common core regulators. However, despite the availability of various animal models, the metabolic pathways underlying the eventual loss of photoreceptors remain largely unknown. Early studies on RP proposed apoptosis as the final common pathway in retinal degeneration (RD) [15], although at that point the idea that apoptosis is just one in a spectrum of cell death principles, and as such hallmarked by caspase activation, was not that clear. Moreover, the previous findings may have been complicated by the fact that in rodent models mutation induced photoreceptor death is often superseded by early postnatal developmental cell death [16]. Indeed, recent evidence collected in the phosphodiesterase-6 (PDE6) mutant *rd1* mouse model for RP suggests the activity of alternative, caspase-independent (and hence non-apoptotic) routes to photoreceptor cell death [17], [18]. It appears obvious that greater knowledge about these neurodegenerative mechanisms is crucial for developing rational therapies.

We studied processes associated with retinal neurodegeneration in the P23H and S334ter rats, with the aim of establishing the metabolic pathways causing photoreceptor cell death in these RP animal models. Intriguingly, a number of caspase-independent processes associated with RD in the *rd1* mouse [16] were also identified in the rhodopsin mutants, suggesting that neurodegenerative pathways triggered by mutations in different genes are very similar across species.

In addition, one of the mutants simultaneously displayed clear signs of conventional, caspase-dependent apoptosis. Our findings are therefore compatible with the idea that alternative cell death mechanisms are, on their own, capable of govern neuronal death in RP. Nevertheless, there are also situations when regular apoptotic pathways are recruited as well. The findings may facilitate future development of mutation-independent neuroprotective therapies in a significant group of ADRP patients.

## Results

### General Morphology – TUNEL staining

Analysis of P23H and S334ter retinal cross-sections during the first postnatal (PN) month showed a progressive reduction in outer nuclear layer (ONL) thickness compared to CD rats as described previously [10], [13]. This was matched by a decrease in the number of photoreceptor rows, which at PN30 showed a ∼40% reduction in P23H mutants (6 ONL cell rows remaining) and 90% in S334ter rats (∼1 row), where remaining ONL cells at PN30 were almost exclusively cones (data not shown).

**Figure 1:**
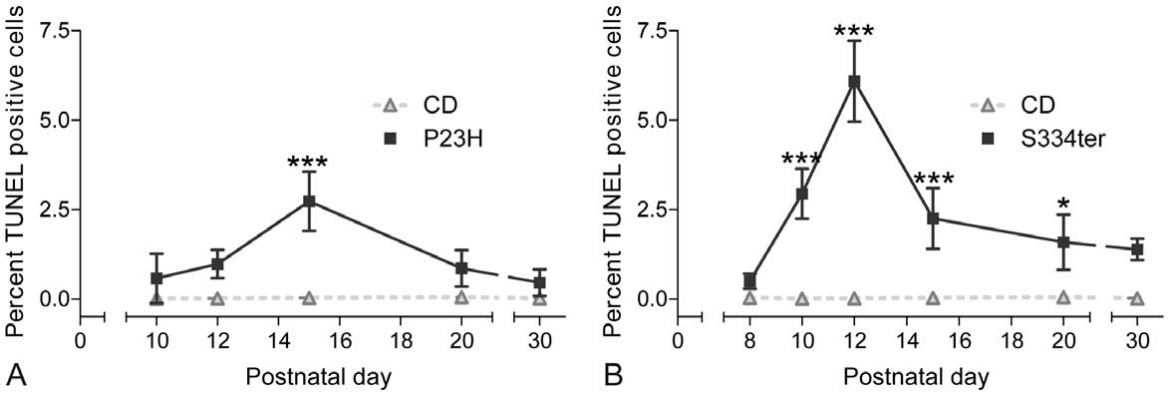
Quantification of photoreceptor cell death during the first postnatal month. (**A**) Increased numbers of TUNEL stained, dying cells in P23H rats, showing a peak of cell death at PN15. (**B**) In S334ter, TUNEL-positive cells were significantly elevated from PN10 onwards, increased in number until PN12 and decreased subsequently. In both models, dying cells were detectable as late as PN30. Values are mean ± SD from at least three different animals; *P<0.05; **P<0.01; ***P<0.001.

**Figure 2:**
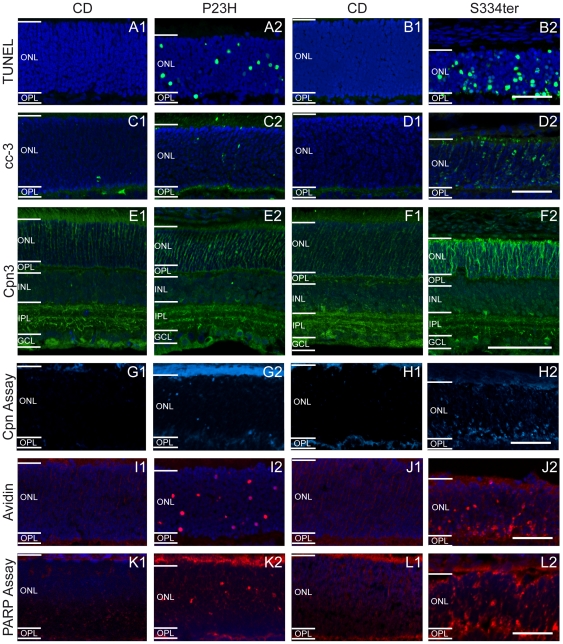
Differential regulation of cell death markers in (1) wt and (2) rhodopsin transgenic rats. (**A–B**) TUNEL assay for dying cells, (**C–D**) caspase-3 immunostaining, (**E–F**) calpain 3 immunostaining, (**G–H**) *in situ* calpain activity assay, (**I–J**) avidin binding and (**K–L**) *in situ* PARP activity assay. Left panels (**A**, **C**, **E**, **G** and **I**) correspond to PN15 animals and right panels (**B**, **D**, **F**, **H** and **J**) to PN12. All stainings showed large numbers of positive cells in P23H and especially in S334ter ONL, but not in wt retina. Scale bar = 50 µm.

We used the TUNEL assay to identify photoreceptors undergoing cell death. Throughout the first PN month, P23H retina demonstrated high levels of TUNEL-positive cells when compared to CD, showing a peak of cell death at PN15 (P23H: 2.7%±0.8 SD, n = 3; CD: 0.02%±0.01 SD, n = 3, *P*<0.001) (Fig. 1A). In S334ter animals, a significant elevation of photoreceptor cell death was evident already at PN10, while the highest percentage of TUNEL-positive ONL cells was found at PN12 (S334ter: 6.1%±1.1 SD, n = 3; CD: 0.02%±0.01 SD, n = 3, *P*<0.001) (Fig. 1B). At PN15, the number of dying ONL cells – while still significantly higher than CD – was declining because by then most photoreceptors had disappeared (S334ter PN15: 2.2%±0.8 SD, n = 3, *P*<0.001). TUNEL-positive cells remained detectable as late as PN30 in both mutants. In CD controls, TUNEL-positive ONL cells were detected only very sporadically (Fig. 1 and Fig. 2A–B; Supporting Table S1).

For a detailed further analysis of other metabolic markers, we chose the ages showing the peak of cell death, i.e. PN15 for P23H and PN12 for S334ter mutants, respectively.

### Activation of caspase-3, caspase-9 and cytochrome c leakage

To study the cell death mechanisms potentially involved in photoreceptor degeneration, we first performed an immunostaining for activated caspase-3, a marker generally used to detect apoptotic cells. Only very few positive cells were found in the ONL of either P23H (0.1%±0.04 SD, *n* = 3) or wild-type (wt) retinas at PN15 (CD: 0.03%±0.02 SD, *n* = 4, *P*>0.05) (Fig. 2C). On the other hand, as previously described [13], activated caspase-3 immunoreactivity was significantly increased in S334ter ONL (S334ter PN12: 5.1%±0.1 SD, *n* = 3) compared to wt control or P23H (CD PN12: 0.01%±0.00 SD, *n* = 3, *P*<0.001; P23H PN12: 0.12%±0.06 SD, *n* = 3, *P*>0.05) (Fig. 2D; Supporting Table S1).

In line with an involvement of apoptosis in the S334ter model only, immunostaining for two other apoptotic markers, caspase-9 cleaved at Asp353 and cytochrome c, also labelled a relatively large number of photoreceptors in S334ter retina, but failed to do so in wt control and P23H rats. (Supporting Fig S3).

### Expression and activity of calpains

Activation of ubiquitously expressed calpain-type proteases has been shown to be involved in neurodegeneration, including in the retina [19], [20] and is often connected to alternative cell death mechanisms [21]. Their involvement in rat rhodopsin mutants has not been described, though. To study a potential participation of calpains in rat RD, we first analysed expression of the major isoforms, calpains-1 to -3. As previously described [22], calpains-1 and -2 were evenly distributed throughout all retinal layers. Neither immunostaining nor WB analysis showed marked differences between P23H or S334ter mutants and their corresponding wt controls (Fig. S1).

Calpain-3 immunostaining was also present in the entire retina, with a more intense labelling observed in a subpopulation of inner nuclear layer and ganglion cell layer cells and in the inner plexiform layer (IPL) in both wt and mutant rats (Fig. 2E–F). Colocalization with choline-acetyl-transferase (ChAT) established more prominent calpain-3 expression in amacrine and horizontal cells and two strata of dendrites in the IPL (Fig. S2). Interestingly, in rhodopsin mutants, ONL calpain-3 immunolabelling was more obvious, with PN12 S334ter retina showing the strongest labelling (Fig. 2F). This staining showed the outline of photoreceptor cells, reflecting a membranous distribution of calpain-3. Nevertheless, WB for calpain-3 failed to detect a significant up-regulation in mutants (data not shown).

**Figure 3:**
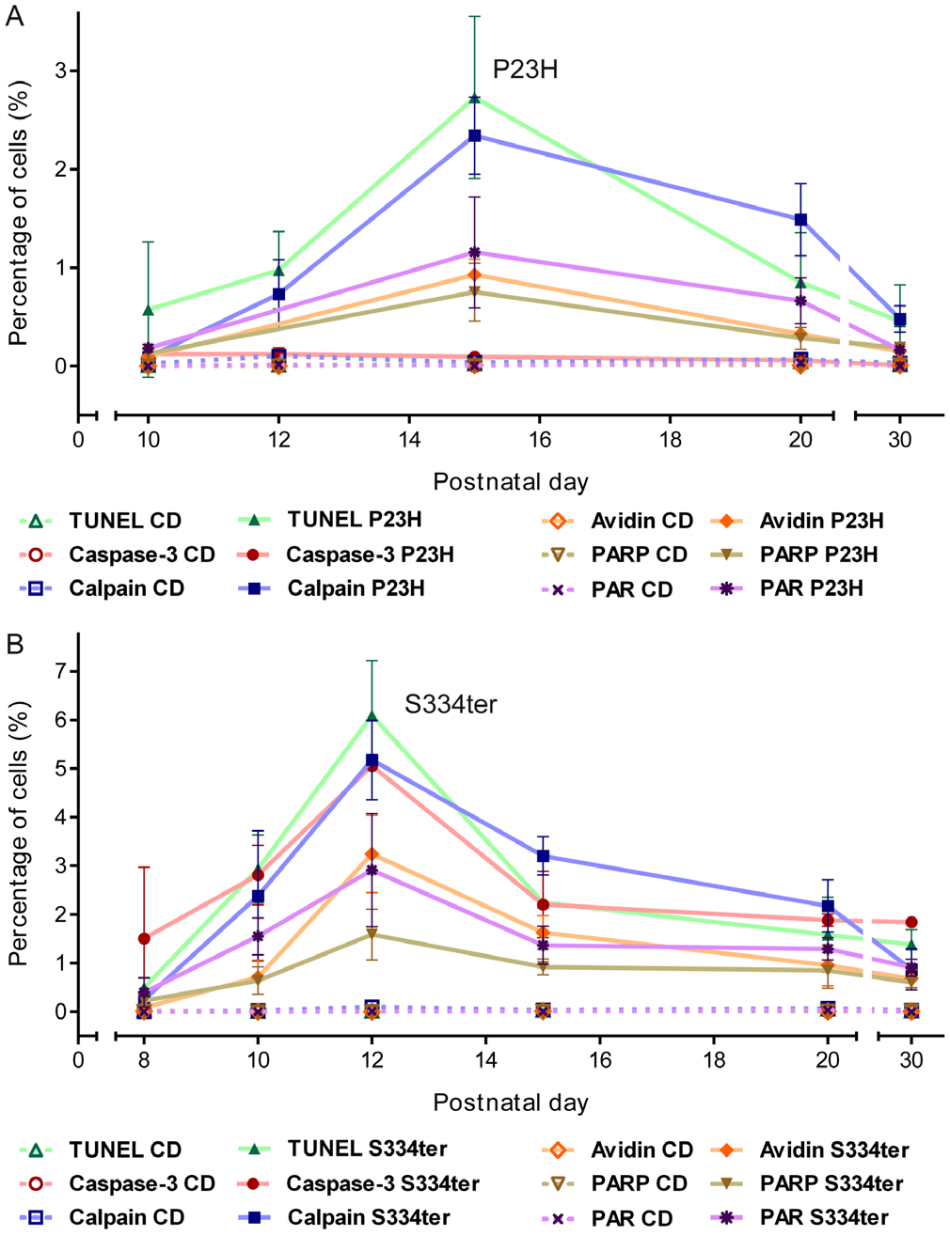
Progression of metabolic cell death markers during 1st postnatal month. Percentage of labelled ONL cells in (**A**) P23H and (**B**) S334ter transgenic rats. While in both RP animal models, most markers analysed peaked together with cell death as evidenced by the TUNEL assay, activation of caspase-3 was absent in P23H retina but present in S334ter retina. In both mutants, calpain activity showed a delayed regression after the peak of cell death. Values are mean ± SD from at least three different animals. All mean ± SD and *P* values are consigned in the table S1.

Since increased calpain activity would not necessarily require increased expression [19], we additionally used an *in situ* assay to study calpain activity directly. In both mutant retinas, numerous photoreceptors were brightly labelled, whereas in wt very few positive cells were detected (Fig. 2G–H). In P23H rats, the number of calpain activity-positive cells was significantly elevated at PN15 (2.3%±0.4 SD, *n* = 4; CD: 0.04%±0.02 SD, *n* = 3, *P*<0.001) (Fig. 3A). Calpain activity in S334ter animals increased progressively reaching a peak value at PN12 (5.2%±0.8 SD, *n* = 4; CD: 0.1%±0.02 SD, *n* = 3, *P*<0.001) (Fig. 3B). The results thus suggest that calpain activation is an integral component in the photoreceptor degeneration mechanism in both the P23H and S334ter rats.

### Calpastatin expression

**Figure 4:**
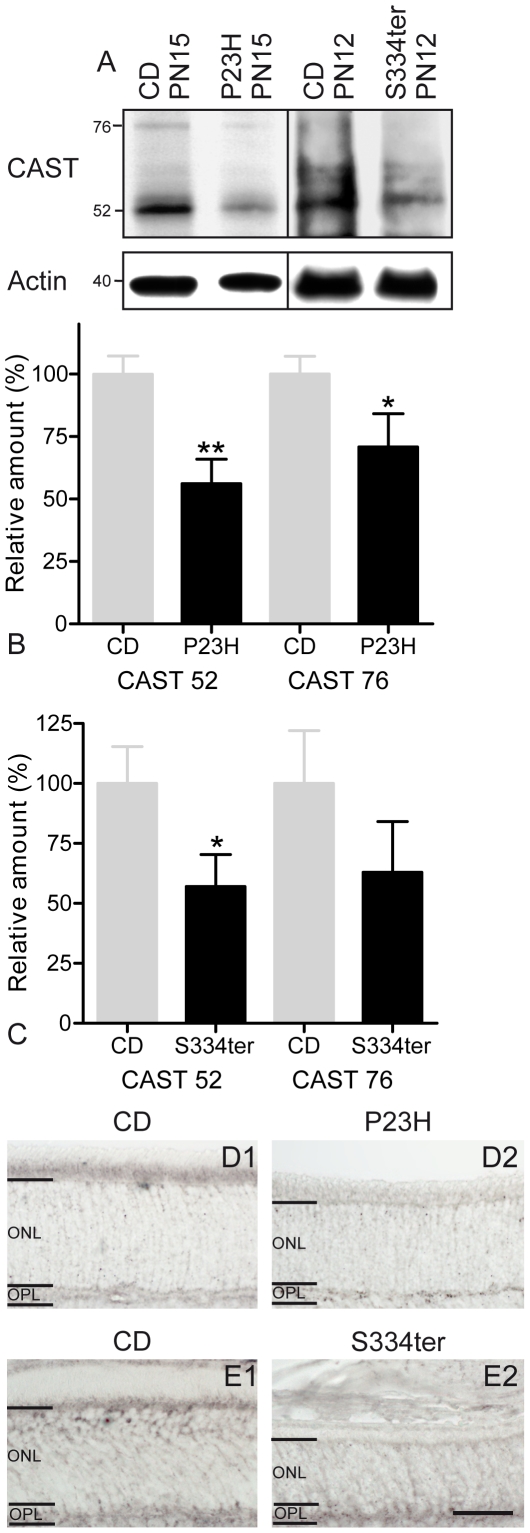
Expression of calpastatin in rhodopsin transgenic rats. (**A**) Immunoblotting for calpastatin showed decreased levels in both, P23H and S334ter rats. (**B–C**) Quantification of the main calpastatin bands at 76 and 52 kDa showed decrease expression in both mutant retinas. This decrease in expression was statistically significant for both bands in (**B**) P23H retina and (**C**) for the 52 kDa band in S334ter retina. Values are mean ± SD from three different experiments each containing retinas from 6 animals. (**D–E**) Calpastatin staining was less intense in inner segments in both rhodopsin transgenic rats. *P<0.05; **P<0.01. Scale bar = 50 µm.

As the activity of calpain is regulated by its endogenous inhibitor calpastatin [23], we investigated its retinal expression. Calpastatin has a predicted molecular weight of ∼77 kDa but WB is known to produce several bands with apparent molecular weights ranging from 17 to 110 kDa and may show considerable variation between different tissues and species [24], [25]. WB identified four major bands corresponding to 52, 60, 65, and 76 kDa (Fig. 4A). Quantification of the main, 52 kDa calpastatin band as well as the expected 76 kDa band and comparison with CD retina showed a statistically significant decrease for both bands in P23H retina (76 kDa: *P*<0.05, 52 kDa: *P*<0.01, *n* = 3) (Fig. 4B), and for the 52 kDa band in S334ter retina (76 kDa: *P*>0.05, 52 kDa: *P*<0.05, *n* = 3) (Fig. 4C).

Calpastatin immunostaining was then performed to test whether the observed decrease in expression at the tissue level was localized to photoreceptors. As described in other species [19], in wt, calpastatin antibody gave a weak labelling of cellular and synaptic retinal layers, with a stronger labelling of photoreceptor inner segments. In both transgenic rats, calpastatin staining was reduced in inner segments, confirming WB results (Fig. 4D–E). A reduction of calpastatin in the mutant photoreceptors may therefore have contributed to the calpain activation in these cells.

### Oxidative DNA damage

Oxidative stress has repeatedly been implicated with photoreceptor degeneration [26] and hence we examined cellular oxidative DNA damage by staining with fluorescently conjugated avidin. Many avidin-positive cells were observed in the ONL of both rhodopsin mutants, with PN12 S334ter retina showing the highest levels of oxidative stress (P23H PN15: 0.9%±0.2 SD, *n* = 3, *P*<0.001; S334ter PN12: 3.2%±0.8 SD, *n* = 3, *P*<0.001) (Fig. 3). In wt, avidin-positive cells were observed only very sporadically (CD PN15: 0.01%±0.01 SD, *n* = 3; PN12: 0.001%±0.002 SD, *n* = 3) (Fig. 2I–J).

### PARP and PAR

An excessive activation of PARP has been found to play a major role in many neurodegenerative diseases [27] and may contribute to caspase-independent photoreceptor death [28]. To investigate PARP activity in transgenic rats, we used two different approaches. First, PARP activity was examined using an *in situ* enzyme assay that detects incorporation of biotin labelled NAD^+^ [29]. Only very few labelled cells were detected in CD ONL, while in P23H (0.8%±0.3 SD, *n* = 5; CD: 0.01%±0.003 SD, *n* = 3, *P*<0.001) and especially in S334ter rats (1.6%±0.5 SD, *n* = 3; CD: 0.004%±0.004 SD, *n* = 3, *P*<0.001), many photoreceptor nuclei were labelled (Fig. 2K–L and Fig. 3).

**Figure 5:**
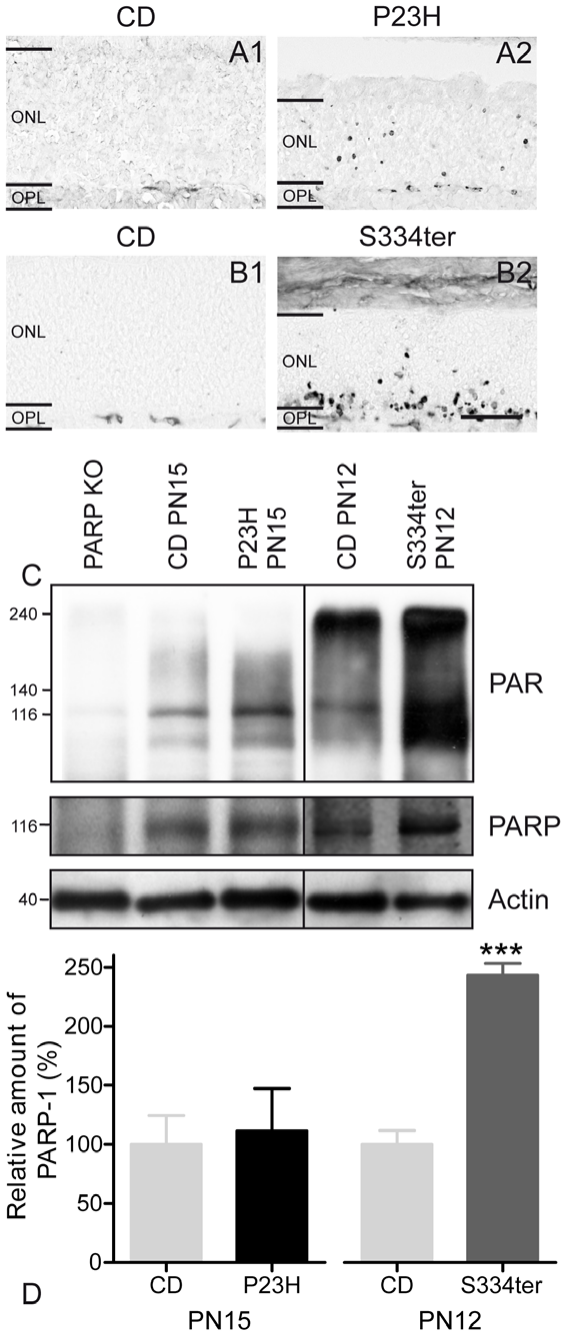
PAR accumulation and PARP-1 expression in rhodopsin transgenic rats. (**A–B**) Accumulation of PAR was found in many cells in P23H and especially in S334ter retinas. (**C**) PAR WB identified a band at ∼116 kDa which most likely represented PARP-1 itself and additionally strong labelling of proteins ranging from 100 to 240 kDa, suggesting poly(ADP-ribosyl)ation. Retinal PAR levels were increased in both transgenic animals. (**C–D**) PARP-1 protein expression was similar in wt and P23H retina, but significantly increased in S334ter. PARP-1 knock-out retina was used as negative control. Values are mean ± SD from four different experiments each containing retinas from 6 animals. ***P<0.001. Scale bar = 50 µm.

Second, we performed PAR immunostaining to test for an accumulation of poly(ADP-ribosyl)ated proteins to indirectly confirm PARP activity. In line with the activity assay results, numerous PAR-positive cells were observed in transgenic rat ONL (Fig. 5A–B), with S334ter retina again showing the highest numbers of positive cells (P23H PN15: 1.2%±0.6 SD, *n* = 3, *P*<0.01; S334ter PN12: 2.9%±1.2 SD, *n* = 3, *P*<0.001). Only very few PAR-positive cells were detected in CD retina (CD PN15: 0.0%±0.0 SD, *n* = 3; CD PN12: 0.01%±0.01 SD, *n* = 3) (Fig. 3). The activity measurements as well as the stainings for its product thus indicated PARP involvement in the degeneration in both mutants.

PAR WB on retinal samples from wt, P23H and S334ter rats showed a strong labelling corresponding to molecular weights ranging from 100–240 kDa. Interestingly, in wt retina, the poly-ADP-ribosylation of high molecular weight proteins appeared to be decreasing with post-natal age. WB also recognized an approximately 116 kDa band which was absent in PARP-1 knock-out mouse samples, suggesting that this band likely indicated auto poly(ADP-ribosyl)ation of PARP-1 [30] (Fig. 5C).

To quantify PARP-1 protein expression levels, WB was used, identifying the characteristic 116 kDa band which was absent in PARP-1 knock-out negative control (Fig. 5C). Quantification showed a statistically significant increase of PARP-1 in S334ter but not in P23H when compared to CD (P23H: *P*>0.05, *n* = 5; S334ter: *P*<0.0001, *n* = 4) (Fig. 5D).

### Differential co-localization and temporal correlation of TUNEL with other metabolic markers

**Figure 6:**
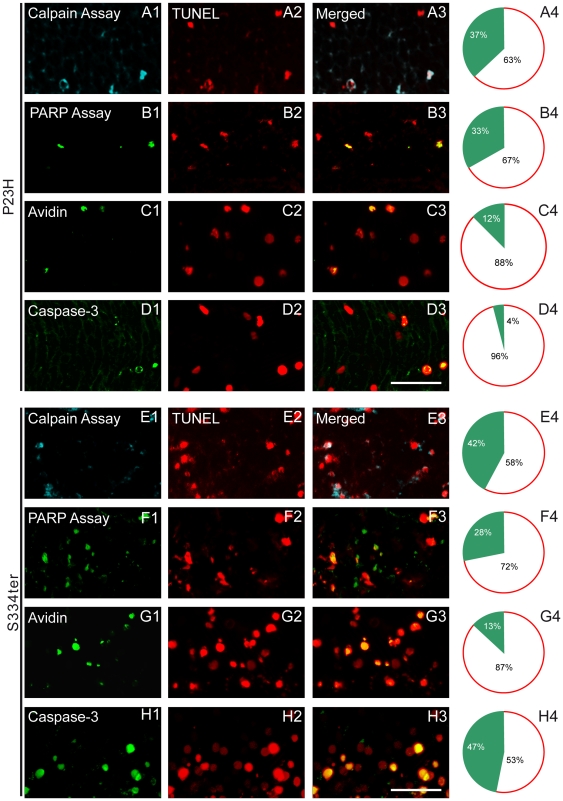
Fluorescence micrographs demonstrating the double-labelling of TUNEL with other metabolic markers. (**A2–H2**) Double labelling of TUNEL with: (**A1**, **E1**) calpain assay, (**B1**, **F1**) PARP assay, (**C1**, **G1**) avidin and (**D1**, **H1**) caspase-3 in (**A–D**) P23H retina at PN15 and (**E–H**) S334ter retina at PN12 transgenic rats. (**A3–H3**) Merged pictures. (**A4–H4**) Right panel indicates the percentages of co-labelled cells in the ONL. In both mutants calpain and PARP activity co-localized with TUNEL in ∼30–40% of cells, while avidin-binding co-localized in 12–13% with TUNEL. Caspase-3 co-localization was observed in almost 47% of TUNEL-positive cells in S334ter but only in 4% in P23H retinas. Scale bar = 25 µm.

To determine the percentage of dying cells labelled for the different biochemical markers, we performed double labelling with TUNEL assay as an indicator for the final stages of cell death. In both mutants, calpain and PARP activity co-localized to a large extent with TUNEL staining (∼30–40%), while avidin labelling for oxidative DNA damage co-localized only in 12–13% of ONL cells (Fig. 6A–C; E–G). Interestingly, caspase-3 co-localization was observed in almost 47% of TUNEL-positive cells in S334ter animals while in P23H retina it, in addition to being very sparse, occurred only in 4% of TUNEL-positive cells (Fig. 6D, H). These results suggest that photoreceptor cell death in both mutants was highly dependent on calpain and PARP activity, with an additional involvement of caspase activity in S334ter but not in P23H retina.

This interpretation is corroborated by a comprehensive analysis of progression over time for the different metabolic markers and TUNEL assay. All studied markers were firmly correlated with the degenerative process, with the exception of caspase-3 activity which was unremarkable in P23H retina (Fig. 3). The complete data sets and respective *P* values are presented in Supporting Table S1.

## Discussion

The neurodegenerative mechanisms governing photoreceptor cell death have remained elusive to date. While a number of early publications suggested an involvement of apoptosis, the present study exposed the fact that markers for conventional apoptotic cell death (i.e. activated caspase-3, caspase-9, cytochrome c leakage) played a role only under specific circumstances and highlighted a significant presence of metabolic markers for non-apoptotic cell death in both rhodopsin mutants. Furthermore, our results suggested that down-stream cell death mechanisms triggered by different genetic mutations, in different species, share a number of key components. These findings will have important implications for the development of mutation-independent RP therapies.

### Caspase activity

Photoreceptor cell death in RD has repeatedly been referred to as apoptosis [15]. The TUNEL method is often used as apoptotic marker even though this assay also detects non-apoptotic DNA fragmentation, e.g. in necrosis [31]. Classical apoptosis depends on activity of caspase-type proteases, with caspase-3 as the prototypic mediator and executioner of apoptotic cell death [32]. Previous studies found caspase-3 activation in the S334ter model [13]. We confirmed these results, however with two important considerations: (i) in S334ter retina, caspase-3 and caspase-9 activity along with cytochrome c leakage appear concomitantly with calpain and PARP activity, indicating that these mechanisms are executed side by side; (ii) in P23H rat, caspase-3 activity was not significantly higher than in age-matched wt suggesting that P23H caspase-3 activity relates to developmental but not to mutation induced cell death. This intriguing discrepancy between the two RP models demonstrates how changes in the location of rhodopsin mutations may determine the phenotype leading to caspase-independent cell death in one case or to degeneration that involves caspase activation in the other.

The activity of caspase-3 independent pathways in both rat mutants and in *rd1* mice [19], [20] may explain why previous experimental approaches using caspase inhibition afforded no or only partial photoreceptor protection [13], [33].

### Calpain activity and Calpastatin expression

Ca^2+^-dependent calpain-type proteases play important roles in many neurodegenerative diseases [34]. In rhodopsin transgenic rats, we did not find obvious expression changes at the tissue level, although the increased visualization of calpain-3 in immunofluorescence may refer to changes in expression, localization, and/or activation within photoreceptors.

Importantly, however, we found strongly elevated calpain activity in rhodopsin transgenic rats matching with a significant decrease in the expression of the endogenous inhibitor calpastatin, which to a large extent regulates the calpains [35], [36]. Our results in rhodopsin mutant retina are in line with earlier studies on *rd1* retina where calpastatin down-regulation corresponds to a strong up-regulation of calpain activity [19] and strengthen the view that calpain activation is an important step in retinal neurodegeneration [16], [19], [20]. Therefore, in the context of neuroprotective treatments, targeting calpain activity may address a larger spectrum of RP-causing mutations and might thus be more effective than inhibiting caspase activity, evident only in the S334ter mutant.

### DNA damage and PARP activity

Oxidative stress has repeatedly been identified as an important contributor to inherited RD [37]. However, at present it is still unclear whether oxidative stress is causally involved in primary RD (i.e. rod degeneration) or whether it is a secondary phenomenon causing mutation-independent death of cones and inner retina neurons [16]. Reactive oxygen species generated for instance by excessive mitochondrial metabolism [38] will create characteristic oxidized compounds, such as 8-oxo-guanosine, the main oxidation product in the DNA [39]. The accumulation of 8-oxo-guanosine observed in photoreceptor nuclei of P23H and S334ter rat retina corresponds to previous findings in *rd1* retina [29], [40]. In both mutant rats and mice oxidative DNA damage may be crucial to trigger activation of PARP, a ubiquitously expressed nuclear protein, which is activated by DNA damage and seen as an important mediator of DNA repair [41]. Nevertheless, an excessive activation of PARP and the production of high levels of neurotoxic PAR polymer [21], [42], have also been connected with cell death, in particular in the context of neurodegenerative diseases, where PARP has been proposed to play a central role in a novel form of caspase-independent cell death, tentatively termed PARthanatos [27].

We found clear evidence for PARP over-activation in degenerating P23H and S334ter photoreceptors, concomitant with an accumulation of PAR, very similar to what was previously observed in the *rd1* mouse [29]. While in P23H rats and *rd1* mice, strong increases in PARP activity were not matched by increased expression, in S334ter retina an up-regulation of both PARP activity and PARP-1 expression was detected. The reasons for this PARP up-regulation exclusively in S334ter retina remain unclear, but may be related to the more severe form of degeneration in this model. However, it does not seem to be a compensatory upregulation due to cleavage by the activated caspase-3, since we saw no signs of increased PARP cleavage product in S334ter tissue. Our data clearly indicates PARP activation as a key step in photoreceptor degeneration regardless of the causal mutation and proposes PARP as a target for neuroprotective treatments.

### Temporal progression of metabolic cell death markers

Both rhodopsin transgenic models are characterized by an early death of photoreceptors, and thus, mutation induced cell death overlapped temporally with developmental cell death [43]. Still, the metabolic markers studied here were strongly increased only in RD mutants, indicating that developmental processes had only a minor, if any, influence. The P23H retina showed a comparatively slower progression of photoreceptor cell death than S334ter, in line with earlier studies [10], [13]. In S334ter rat, caspase-3 activation was elevated, however, it coincided numerically and temporally with calpain activation during the second PN week, indicating that these two proteolytic systems are activated and executed in parallel. This co-activation suggests some form of cross-talk between caspases and calpains [44] and may explain at least partially, the extremely fast progression of degeneration in this model. Alternatively, it would also be possible that a different cell death mechanism was triggered because of the faster progression of S334ter degeneration (possibly linked to a stronger genetic insult). However, in the *rd1* mouse, which displays essentially the same rate of cell death, only non-apoptotic markers are observed [15], suggesting that the cell death pathway was not *per se* determined by the speed of degeneration.

In P23H retina, caspase-3 activity clearly plays only a minor, if any, role while at the same time calpain activity is very prominent. The implications for a therapy of RP in this model are important since these results suggest that targeting exclusively the caspase cascade – as was previously proposed for the *rd1* mouse [33] - is unlikely to give positive results while, on the other hand, targeting non-apoptotic events such as calpain or PARP activation may have beneficial effects [20], [28]. A detailed identification of all associated and interacting proteins involved in photoreceptor cell death is fundamental, because if a single enzyme is blocked, the cell may still be able to activate/continue other mechanisms or routes to die [44], [45].

Finally, the increase of all analysed markers (i.e. caspase-3 activation, calpain and PARP activity, PAR accumulation, oxidative DNA damage, TUNEL) coincided and followed a similar pattern. The metabolic markers correlated temporally and even co-localized with TUNEL staining, proposing that these events occurred relatively late in the degenerative process. Alternatively, this close correlation may also be explained when assuming that cell death, once triggered, is executed very rapidly. Remarkably, in both transgenic models, after the peak of degeneration, the number of cells showing calpain activity exceeded the number of TUNEL-positive cells. This could be due to a higher detection sensitivity of the calpain assay, but may also indicate that proteolytic activity of calpains persists at times when the nuclear DNA has already disintegrated.

In conclusion, we here provide for the first time a comprehensive overview of several important metabolic markers associated with photoreceptor cell death in two mutant rhodopsin models for RP. Furthermore, we highlight differences and similarities between the two models, as well as between different rodent species. While in S334ter retina, cell death was associated with both apoptotic and non-apoptotic markers, P23H mutation induced cell death appeared to be restricted to non-apoptotic mechanisms. Interestingly, in both mutants, cell death was clearly connected to activation of calpain and PARP, as well as accumulation of PAR and oxidatively damaged DNA, similar to what has previously been observed in *rd1* mouse retina. Taken together, these findings suggest that non-apoptotic cell death plays an important role in inherited photoreceptor degenerations, in general, while apoptosis may additionally occur in certain mutations such as rhodopsin C-terminal truncations.

This in turn may have important implications for future therapy development because (i) it expands the perimeter from apoptotic to other, alternative mechanisms which in turn may yield a number of novel targets for neuroprotective treatments (ii) the same non-apoptotic processes were observed in the two rhodopsin mutants as well as in the PDE6 mutant *rd1* mouse and hence strongly improve the perspectives for a mutation-independent neuroprotective treatment of RP.

## Materials and Methods

### Ethic statement

All procedures were approved by the Tuebingen University committee on animal protection and performed in compliance with the ARVO statement for the use of animals in Ophthalmic and Visual Research. Protocols compliant with § 4 paragraph 3 of the German law on animal protection were reviewed and approved by the “Einrichtung für Tierschutz, Tierärztlichen Dienst und Labortierkunde” (Anzeige/Mitteilung nach § 4 vom 28.04.08 and 29.04.10). All efforts were made to minimize the number of animals used and their suffering.

### Animals

Homozygous P23H and S334ter rhodopsin transgenic rats (produced by Chrysalis DNX Transgenic Sciences, Princeton, NJ) of the line Tg(P23H)1Lav and Tg(S334ter)3Lav (P23H-1 and S334ter-3) were kindly provided by Dr. M. M. LaVail (University of California, San Francisco, CA) and bred in our animal housing facility. We employed heterozygous P23H and S334ter rats, obtained by crossing with wt, CD rats (CD® IGS Rat; Charles River, Germany) to reflect the genetic background of ADRP. CD animals were used as wild type (wt) controls.

### Histology

Rats were euthanized at different ages (PN8–PN30). Fixed (4% paraformaldehyde (PFA) in 0.1 M phosphate buffer (pH 7.4) for 30 min at 4°C and unfixed eyecups were embedded in cryomatrix (Tissue-Tek, Leica, Bensheim, Germany). Radial 12 µm sections were stored at −20°C.

### TUNEL Assay

Terminal deoxynucleotidyl transferase dUTP nick end labeling (TUNEL) assay was performed using an *in situ* cell death detection kit (Fluorescein or TMR; Roche Diagnostics GmbH, Mannheim, Germany). For co-localization with calpain or poly(ADP-ribose) polymerase activity, the activity-stained sections were fixed in 4% PFA and the TUNEL staining was performed afterwards. For co-localization with cleaved caspase-3 or avidin, staining was followed by TUNEL staining.

### Immunohistochemistry

Sections were incubated overnight at 4°C with primary antibodies (Table 1). Immunostaining was performed employing the avidin-biotin-peroxidase technique (Vectastain ABC system, Vector laboratories, Burlingame, CA). Fluorescence immunocytochemistry was performed using Alexa Fluor® 488 conjugated secondary antibody (Molecular Probes, Inc. Eugene, USA). Negative controls were carried out by omitting the primary antibody.

**Table 1:** List of antibodies used in this study.

*Antigen*	*Source/Cat. Number*	*Dilution*	
		*IF/IHC*	*WB*
Calpastatin	Novus Biologicals/NB120-5582	1∶50	1∶5000
Cleaved Caspase-3 (Asp175) (5A1E)	Cell Signalling/9664	1∶300	-
Cleaved Caspase-9 (Asp353)	Abcam/ab52298	1∶100	-
Calpain LP85 and LP82 (Capn3)	Millipore Chemicon/AB81011	1∶50	1∶5000
m-Calpain, large (catalytic) subunit (Capn2)	Millipore Chemicon/AB81023	1∶100	1∶1000
μ-Calpain large subunit, clone P-6 (Capn1)	Millipore Chemicon/MAB3082	1∶100	1∶1000
Choline Acetyltransferase (ChAT; clone 1E6)	Chemicon/MAB 305	1∶300	-
Cytochrome C (clone 7H8.2C12)	BD Pharmingen/556433	1∶2000	-
Poly (ADP-Ribose) Polymerase (PARP; clone C2-10)	BD Pharmingen/556362	-	1∶1000
PAR (10H)	Enzo/ALX-804-220	1∶200	1∶1000

### Calpain activity Assay

Calpain activity was investigated with an enzymatic *in situ* assay [19]. Briefly, unfixed cryosections were incubated for 15 min in calpain reaction buffer (CRB; 25 mM HEPES, 65 mM KCl, 2 mM MgCl_2_, 1,5 mM CaCl_2_, 2 mM DTT) and then sections were incubated at 35°C for 1 h in the dark in 2 µM fluorescent calpain substrate 7-amino-4-chloromethylcoumarin, t-BOC-L-leucyl- L-methionine amide (CMAC, t-BOC-Leu-Met; Molecular Probes, Inc. Eugene, USA). Fluorescence was generated by calpain-dependent cleavage of t-Boc-Leu-Met-CMAC.

### Histology for oxidative DNA damage: Avidin Staining

Avidin staining can be used to identify oxidatively damaged DNA, since it labels 8-OHdG (8-oxoguanine) [40], [46]. Retinal sections were incubated at room temperature for 1 h with Alexa Fluor® 488 conjugated avidin (1∶80). Negative controls were treated with avidin staining solution pre-adsorbed with 12 nM 8-Hydroxy-2′-deoxyguanosine (Calbiochem, Merck KGaA, Darmstadt, Germany) and 50 nM biotin (Vector laboratories, Burlingame, CA).

### Poly(ADP-ribose) polymerase (PARP) enzyme activity assay

Unfixed cryosections were incubated in a avidin/biotin blocking kit (Vector Laboratories, Burlingame, USA), followed by incubation at 37°C for 2 h in PARP reaction mixture containing 10 mM MgCl_2_, 1 mM DTT, 5 µM biotinylated NAD^+^ (Trevigen, Gaithersburg, USA) in 100 mM Tris buffer with 0.2% Triton X-100 (pH 8.0). Incorporated biotin was detected by avidin, Alexa Fluor® 488 conjugate (1∶800, 1 h at room temperature). For controls biotinylated NAD^+^ was omitted from the reaction mixture [29].

### Western Blot (WB)

Retinas were homogenized in buffer (20 mM Tris-HCl (pH 7.4), 0.25 M sucrose, 1 mM EDTA, 0.5 g/L BSA, and 100 µM phenylmethylsulfonyl fluoride [PMSF]) supplemented with protease inhibitors (Set III; Calbiochem, Schwalbach, Germany). Samples were mixed with Laemmli SDS-PAGE sample buffer, boiled for 5 min, separated on 10% SDS-polyacrylamide gels, and electrotransferred to PVDF membranes (Bio-Rad, München, Germany). Membranes were blocked with blocking solution (Roti®-Block, Carl Roth, Karlsruhe, Germany) and incubated overnight at 4°C with primary antibody (see table 1) diluted in TBS-T buffer (150 mM NaCl, 13 mM Tris-HCl [pH 7.5], 0.02% Triton-X-100) or PBS-T (0.1% Tween in case of PARP-1 and PAR) with 5% dry milk. The reaction was visualized with horseradish peroxidase–conjugated secondary antibody and chemiluminescence reagent (ECL Plus; Amersham). Quantification of relative WB band intensities was done following a tutorial written by Luke Miller (http://lukemiller.org/index.php/2010/11/analyzing-gels-and-western-blots-with-image-j/).

### Microscopy, cell counting, and statistical analysis

Light and fluorescence microscopy was performed on an Axio Imager Z1 ApoTome Microscope, equipped with a Zeiss Axiocam digital camera. Images were captured using Zeiss Axiovision 4.7 software; representative pictures were taken from central areas of the retina. Adobe Photoshop CS3 (Adobe Systems Incorporated, San Jose, CA) was used for primary image processing.

For cell quantifications, pictures were captured of whole radial slices using Mosaix mode of Axiovision 4.7 at 20× magnification. Labelled cells were counted manually. The total number of cells was determined by dividing outer nuclear layer (ONL) area through average cell size. The number of positive cells was then divided by the total number of ONL cells giving the percentage of positive cells. All data given represent the means and standard deviations from three sections each, for at least three different animals. Statistical comparisons between experimental groups were made using one-way ANOVA and Bonferroni's correction using Prism 5 for Windows (GraphPad Software, La Jolla, CA).

## Supporting Information

Figure S1 **Calpain 1 and 2 immunolabelling in rhodopsin transgenic rats.** Antibodies directed against (**A–B**) calpain 1 and (**C–D**) calpain 2 are evenly distributed throughout all retinal layers. Immunostaining did not show differences between (**A2, C2**) P23H at PN15 or (**B2, D2**) S334ter at PN12 mutants and (**A1, B1, C1 and D1**) their corresponding wt controls. Scale bar 100 µm.
(TIF)

Figure S2 **Calpain 3 immunolabelling in rhodopsin transgenic rats.** Double staining with an antibody directed against Choline-acetyl-transferase (ChAT) shows that calpain-3 is expressed in cholinergic amacrine cells, horizontal cells and the two strata of dendrites in the IPL. Scale bar 25 µm.
(TIF)

Figure S3 **Caspase-9 cleaved ASP353 and cytochrome c immunolabelling in rhodopsin transgenic rats.** Both stainings showed large numbers of positive cells in S334ter ONL, but not in wt or P23H retina. Scale bar = 50 µm.
(TIF)

Table S1 **Percentage of labelled cells for each cell death marker.**
(DOC)
