# Supplementary figures and images for: Calpain and PARP Activation during Photoreceptor Cell Death in P23H and S334ter Rhodopsin Mutant Rats
Source: PLoS One. 2011 Jul 12;6(7):e22181. doi: 10.1371/journal.pone.0022181 (PMC3134478; doi:10.1371/journal.pone.0022181)

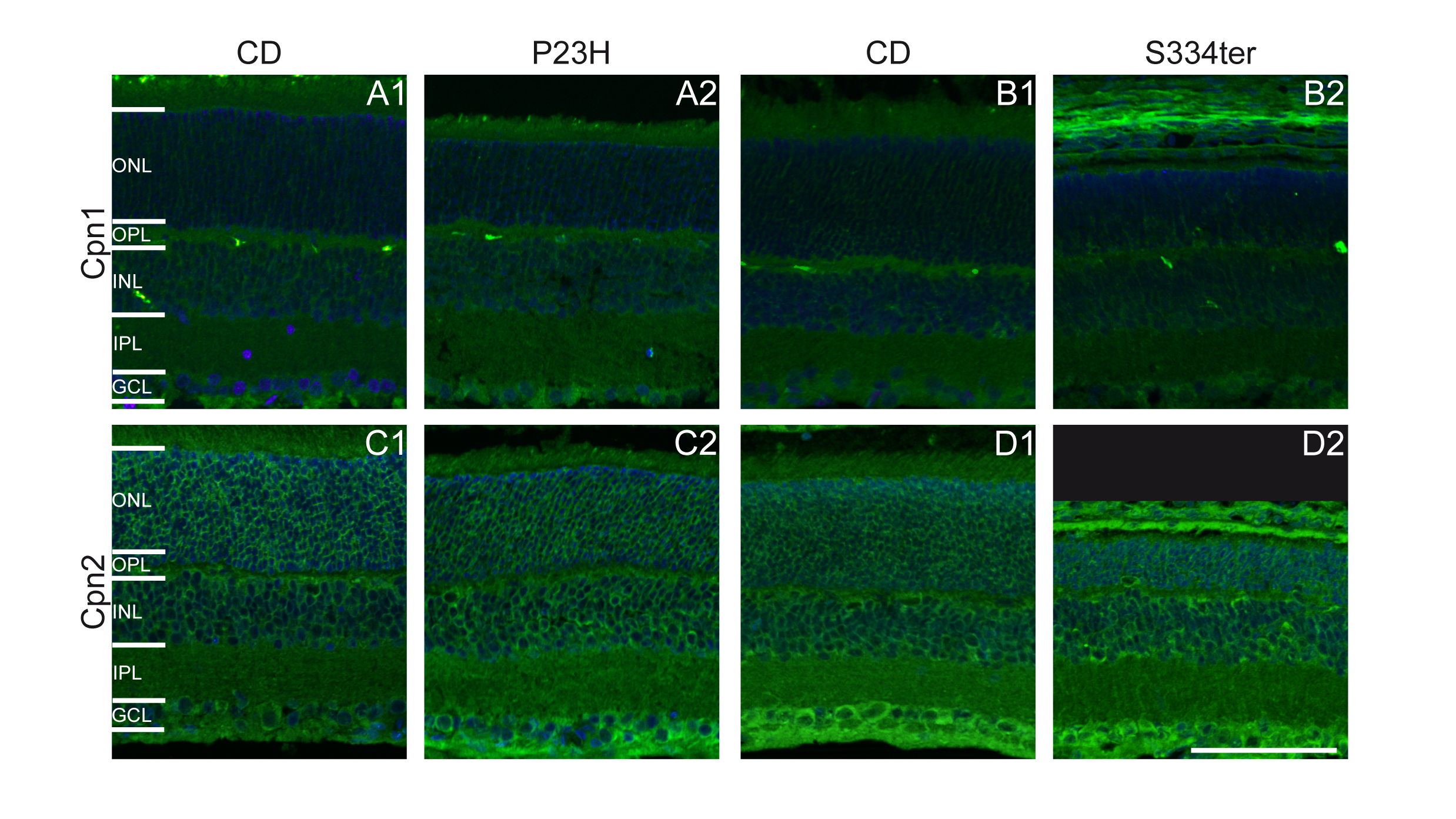

Supplement: Figure S1 — Calpain 1 and 2 immunolabelling in rhodopsin transgenic rats. Antibodies directed against (A–B) calpain 1 and (C–D) calpain 2 are evenly distributed throughout all retinal layers. Immunostaining did not show differences between (A2, C2) P23H at PN15 or (B2, D2) S334ter at PN12 mutants and (A1, B1, C1 and D1) their corresponding wt controls. Scale bar 100 µm. (TIF) [file pone.0022181.s001.tif]

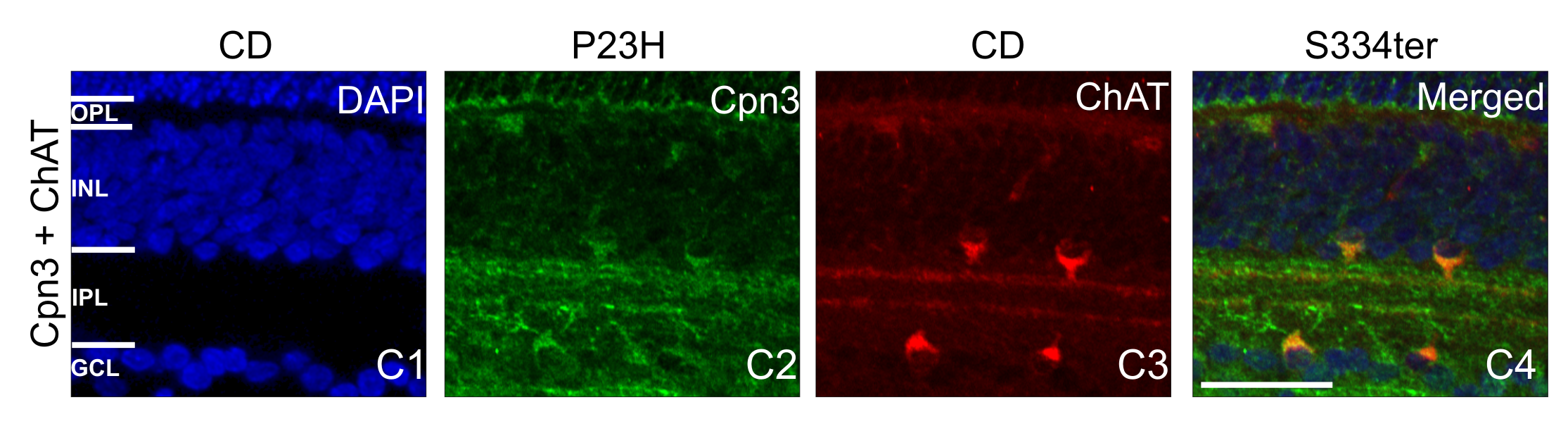

Supplement: Figure S2 — Calpain 3 immunolabelling in rhodopsin transgenic rats. Double staining with an antibody directed against Choline-acetyl-transferase (ChAT) shows that calpain-3 is expressed in cholinergic amacrine cells, horizontal cells and the two strata of dendrites in the IPL. Scale bar 25 µm. (TIF) [file pone.0022181.s002.tif]

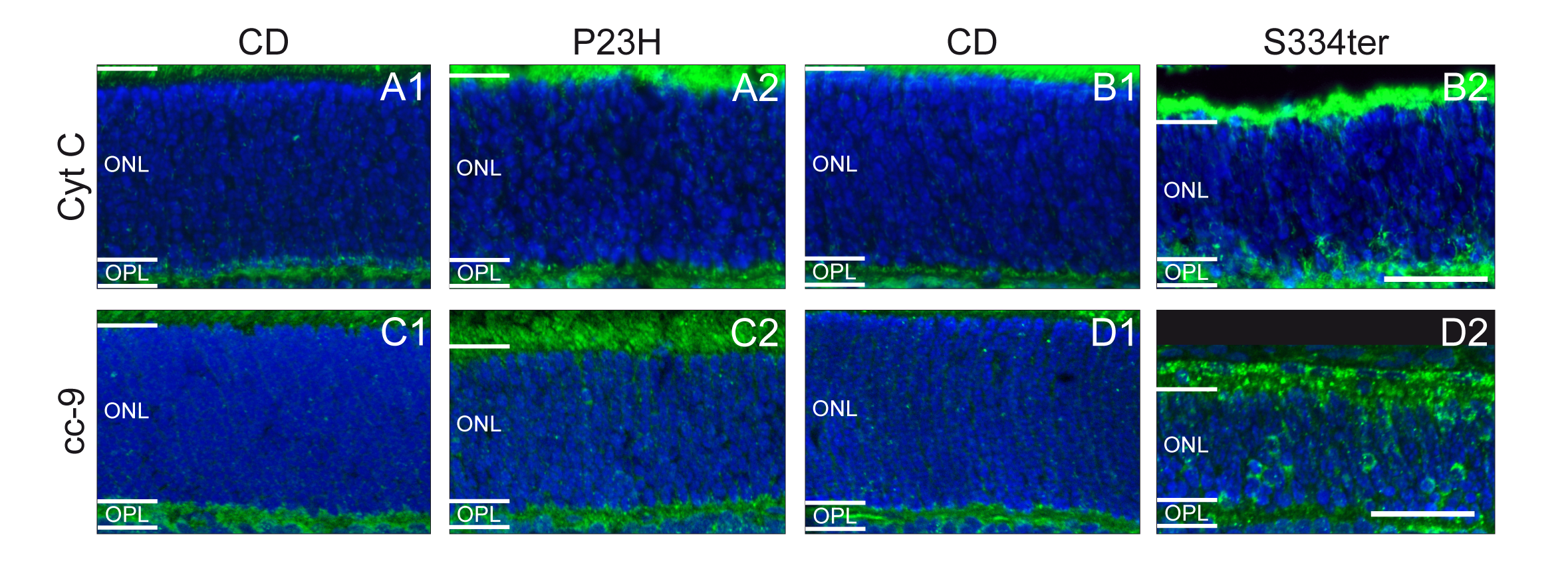

Supplement: Figure S3 — Caspase-9 cleaved ASP353 and cytochrome c immunolabelling in rhodopsin transgenic rats. Both stainings showed large numbers of positive cells in S334ter ONL, but not in wt or P23H retina. Scale bar = 50 µm. (TIF) [file pone.0022181.s003.tif]
