# Supplementary material for: Calpain and PARP Activation during Photoreceptor Cell Death in P23H and S334ter Rhodopsin Mutant Rats
Source: PLoS One. 2011 Jul 12;6(7):e22181. doi: 10.1371/journal.pone.0022181 (PMC3134478; doi:10.1371/journal.pone.0022181)
Supplement: Table S1 — Percentage of labelled cells for each cell death marker. (DOC) [file pone.0022181.s004.doc]

**Supporting Table S1.** Percentage of labelled cells for each cell death marker.

| Age | Animal | TUNEL | | Caspase-3 | | Calpain assay | | Avidin | | PARP | | PAR | |
| --- | --- | --- | --- | --- | --- | --- | --- | --- | --- | --- | --- | --- | --- |
|  |  | Mean ± SD | *p* | Mean ± SD | *p* | Mean ± SD | *p* | Mean ± SD | *p* | Mean ± SD | *p* | Mean ± SD | *p* |
| PN8 | CD | 0,026 ± 0,044 |  | 0,014 ± 0,006 |  | 0,003 ± 0,004 |  | 0,009 ± 0,008 |  | 0,004 ± 0,006 |  | 0,000 ± 0,000 |  |
|  | S334ter | 0,493 ± 0,209 | >0.05 | 1,504 ± 1,469 | <0.01 | 0,245 ± 0,132 | >0.05 | 0,083 ± 0,116 | >0.05 | 0,216 ± 0,103 | >0.05 | 0,394 ± 0,294 | >0.05 |
| PN10 | CD | 0,004 ± 0,003 |  | 0,003 ± 0,002 |  | 0,025 ± 0,013 |  | 0,000 ± 0,000 |  | 0,001 ± 0,002 |  | 0,000 ± 0,000 |  |
|  | P23H | 0,573 ± 0,687 | >0.05 | 0,121 ± 0,102 | >0.05 | 0,088 ± 0,007 | >0.05 | 0,095 ± 0,057 | >0.05 | 0,125 ± 0,059 | >0.05 | 0,181 ± 0,053 | >0.05 |
|  | S334ter | 2,939 ± 0,698 | <0.001 | 2,811 ± 0,614 | <0.001 | 2,381 ± 1,339 | <0.001 | 0,710 ± 0,347 | <0.05 | 0,639 ± 0,280 | <0.01 | 1,549 ± 0,379 | <0.001 |
| PN12 | CD | 0,015 ± 0,012 |  | 0,007 ± 0,004 |  | 0,101 ± 0,015 |  | 0,001 ± 0,002 |  | 0,004 ± 0,004 |  | 0,010 ± 0,011 |  |
|  | P23H | 0,974 ± 0,392 | >0.05 | 0,121 ± 0,065 | >0.05 | NA |  | NA |  | NA |  | NA |  |
|  | S334ter | 6,088 ± 1,132 | <0.001 | 5,056 ± 0,115 | <0.001 | 5,176 ± 0,818 | <0.001 | 3,245 ± 0,793 | <0.001 | 1,585 ± 0,523 | <0.001 | 2,910 ± 1,161 | <0.001 |
| PN15 | CD | 0,023 ± 0,004 |  | 0,031 ± 0,016 |  | 0,036 ± 0,024 |  | 0,009 ± 0,007 |  | 0,011 ± 0,003 |  | 0,000 ± 0,000 |  |
|  | P23H | 2,729 ± 0,824 | <0.001 | 0,094 ± 0,044 | >0.05 | 2,342 ± 0,392 | <0.001 | 0,928 ± 0,158 | <0.001 | 0,751 ± 0,293 | <0.001 | 1,155 ± 0,563 | <0.01 |
|  | S334ter | 2,245 ± 0,846 | <0.001 | 2,205 ± 0,683 | <0.001 | 3,205 ± 0,393 | <0.001 | 1,628 ± 0,347 | <0.001 | 0,921 ± 0,165 | <0.001 | 1,365 ± 0,393 | <0.001 |
| PN20 | CD | 0,045 ± 0,035 |  | 0,011 ± 0,010 |  | 0,070 ± 0,068 |  | 0,005 ± 0,004 |  | 0,014 ± 0,005 |  | 0,033 ± 0,056 |  |
|  | P23H | 0,850 ± 0,508 | >0.05 | 0,059 ± 0,070 | >0.05 | 1,488 ± 0,368 | <0.01 | 0,324 ± 0,025 | >0.05 | 0,282 ± 0,110 | >0.05 | 0,663 ± 0,231 | >0.05 |
|  | S334ter | 1,583 ± 0,771 | <0.05 | 1,881 ± 0,126 | <0.001 | 2,175 ± 0,536 | <0.001 | 0,952 ± 0,422 | <0.001 | 0,846 ± 0,366 | <0.001 | 1,292 ± 0,228 | <0.01 |
| PN30 | CD | 0,013 ± 0,022 |  | 0,000 ± 0,000 |  | 0,030 ± 0,046 |  | 0,006 ± 0,011 |  | 0,017 ± 0,017 |  | 0,000 ± 0,000 |  |
|  | P23H | 0,453 ± 0,372 | >0.05 | 0,007 ± 0,000 | >0.05 | 0,479 ± 0,133 | >0.05 | 0,149 ± 0,072 | >0.05 | 0,186 ± 0,022 | >0.05 | 0,163 ± 0,024 | >0.05 |
|  | S334ter | 1,382 ± 0,303 | >0.05 | 1,840 ± 0,000 | <0.01 | 0,869 ± 0,424 | >0.05 | 0,677 ± 0,186 | <0.05 | 0,604 ± 0,075 | <0.01 | 0,908 ± 0,173 | >0.05 |
